# Statins for the prevention of proliferative vitreoretinopathy: cellular responses in cultured cells and clinical statin concentrations in the vitreous

**DOI:** 10.1038/s41598-020-80127-1

**Published:** 2021-01-13

**Authors:** Yashavanthi Mysore, Eva M. del Amo, Sirpa Loukovaara, Marja Hagström, Arto Urtti, Anu Kauppinen

**Affiliations:** 1 School of Pharmacy, Faculty of Health Sciences, University of Eastern Finland, Yliopistonranta 1C, P.O.B. 1627, 70211 Kuopio, Finland; 2 Department of Ophthalmology, Unit of Vitreoretinal Surgery, Helsinki University Central Hospital, and Individualized Drug Therapy Research Program, University of Helsinki, Helsinki, Finland; 3 School of Pharmacy, University of Helsinki, Helsinki, Finland; 4 Institute of Chemistry, St. Petersburg State University, Petergof, Russian Federation

**Keywords:** Biochemistry, Eye diseases, Inflammation, Eye manifestations, Cytokines, Inflammation

## Abstract

Proliferative vitreoretinopathy (PVR) with rhegmatogenous retinal detachment (RRD) is a complex inflammatory ocular disease. Statins are widely used cholesterol-lowering drugs with putative anti-inflammatory properties. In this study, we have explored their efficacy in controlling post-surgical PVR formation. Simvastatin (SIM), atorvastatin (ATV), or rosuvastatin (RSV) were added to cultures of human retinal pigment epithelial cells (ARPE-19) prior to exposure with the bacterial lipopolysaccharide (LPS), and the production of pro-inflammatory cytokines (IL-6, IL-8, MCP-1) was examined using an enzyme-linked immunosorbent assay. In addition, the concentrations of simvastatin, atorvastatin, rosuvastatin, and their metabolites were measured from the vitreal samples of 20 patients undergoing vitrectomy (16 of them receiving oral statin therapy) using an ultra-performance liquid chromatography-tandem mass spectrometer technique. All statins alleviated LPS-induced inflammation at 5 µM concentration in the ARPE-19 cell cultures. Statin levels in the vitreous samples ranged from 6 to 316 pg/mL (ca. 0.1–7 M^−10^). Vitreal statin concentrations were similar to the typical steady-state unbound statin concentrations in plasma, indicating that only the unbound drug distributes from the blood circulation into the vitreous. Pharmacokinetic simulations of the intravitreal delivery of statins indicate that the measured clinical statin concentrations could be maintained with existing drug delivery technologies for months. Our results suggest that intravitreal statin therapy may have the potential in alleviating the risk of post-surgical PVR.

## Introduction

Rhegmatogenous and tractional retinal detachment (RRD/TRD), separation of the neurosensory retina from its underlying retinal pigment epithelium (RPE), are potentially sight-threatening vitreoretinal conditions associated with mechanisms related to inflammation and hypoxia. RPE cells respond to stress by initiating inflammation, which is followed by the recruitment of neutrophils and blood monocytes that differentiate into macrophages and dendritic cells when these cells gain access to the inflamed tissue^1^. If the inflammatory response in RRD is not quickly controlled, it can result in proliferative vitreoretinopathy (PVR), a pathological process associated with a cytokine and chemokine storm^2–4^, potentially leading to the death of photoreceptor cells and the loss of vision^5^. Major pro-inflammatory factors, such as interleukin 6 (IL-6), neutrophil chemotactic factor (IL-8), and monocyte chemoattractant protein-1 (MCP-1), are released in the early phase of the inflammation in the RRD eyes^6,7^. IL-6 and IL-8 have been associated with the development of PVR as have the functions of extracellular matrix metalloproteinases (MMP-2, MMP-9)^3,7–9^. Despite intense efforts, no efficacious treatments are available to reduce the risk of PVR development in eyes with RRD^10^.

Statins, i.e. β-Hydroxy β-methylglutaryl-CoA (HMG-CoA) reductase inhibitors, are per oral lipid-lowering drugs which have been claimed to exert anti-inflammatory and neuroprotective properties in the RRD eyes as well as in diabetic and neurodegenerative posterior segment eye disorders^11–14^. They have also shown beneficial effects in the retinal-wound healing process, which is related to PVR formation^12^. In rats, simvastatin has improved retinal neuronal cell survival in an experimental ischemia–reperfusion injury^15^ and inhibited experimental PVR in rabbits^16^. In in vitro experiments*,* it also inhibited the collagen gel contractility of cultured hyalocytes from human patients with PVR and proliferative diabetic retinopathy (PDR)^16^. Clinically, the use of simvastatin was associated with a reduced risk of re-vitrectomy in patients who had undergone previous RRD surgery^12^.

The effects of statins are predicted to depend on their dosing and exposure, their lipophilic/hydrophilic properties, blood-ocular barrier permeation, and other aspects of their pharmacokinetics^17,18^. Even though statins have been shown to cross the blood-retinal barrier in a preclinical study^19^, no clinical pharmacokinetic data are available on statin concentrations in the vitreous during per oral statin therapy. In addition, although the mechanisms by which statins could act in the prevention of PVR development have been speculated^10,16^ they have not been studied in detail. Here, we explored the anti-inflammatory effects of simvastatin, atorvastatin, and rosuvastatin on human RPE cells. Moreover, the concentrations of per orally administered simvastatin, atorvastatin and rosuvastatin in the vitreous humour of patients with vitrectomy operation were determined and pharmacokinetically analysed.

## Results

### Predicted lipophilicity and intravitreal clearance of statins: simvastatin > atorvastatin > rosuvastatin

The chemical structures and some relevant physicochemical descriptors of simvastatin, atorvastatin, and rosuvastatin are presented in Table 1. The manufacturer stated that all statins are to be dissolved in dimethyl sulfoxide (DMSO). First, we wanted to analyse whether there would be differences in their lipophilicity. Increased lipophilicity is characterized as having high values of the logarithm of the octanol–water distribution coefficient at pH 7.4 (LogD_7.4_); in our examples, the order of lipophilicity was simvastatin > atorvastatin > rosuvastatin (Table 1).

**Table 1:**
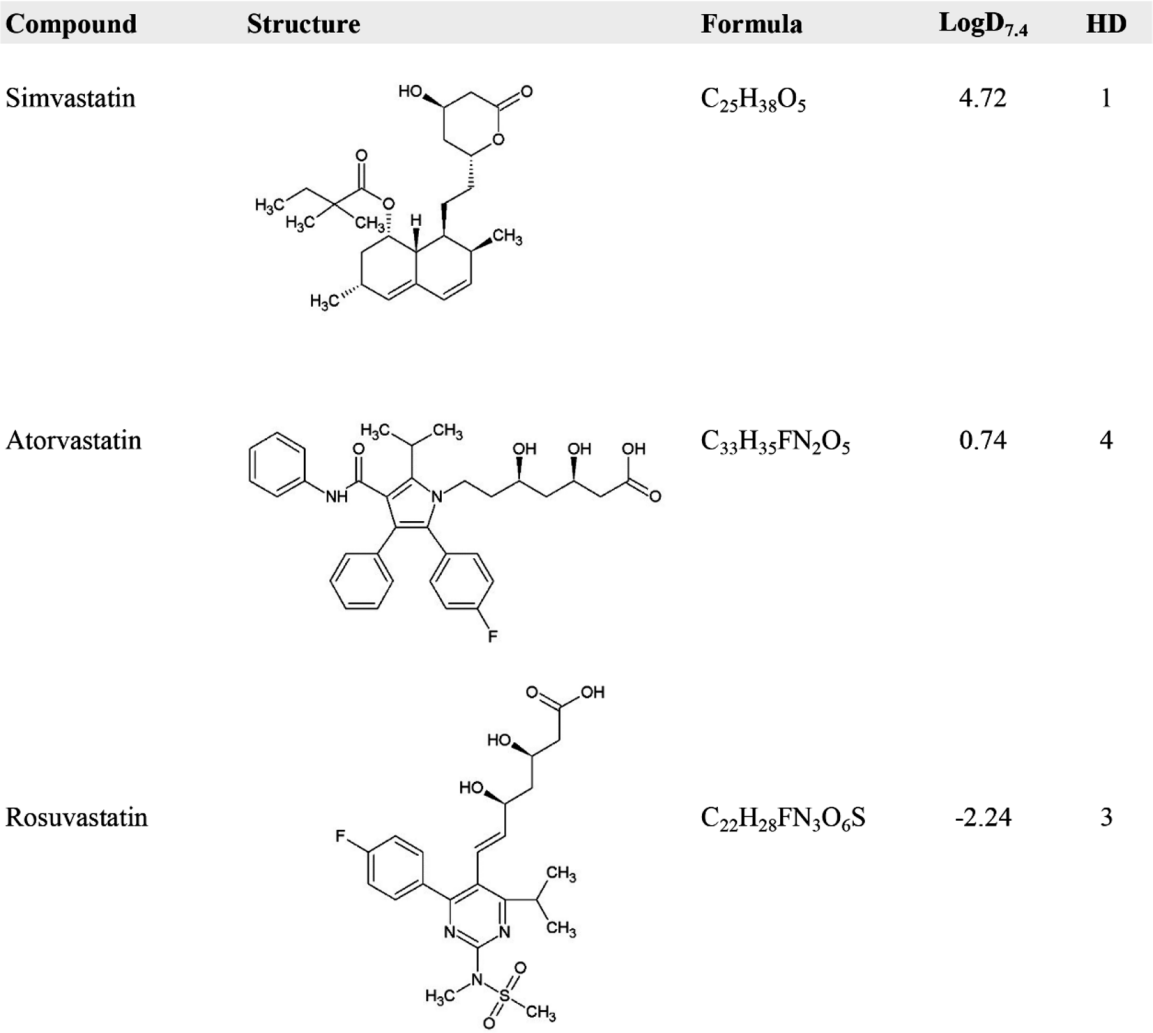
The chemical structures of simvastatin, atorvastatin, and rosuvastatin and the corresponding physicochemical descriptors of Log D_7.4_ and HD.

Rabbit intravitreal clearance (CL_ivt_) can be described based on hydrogen bonding (HD) and lipophilicity (LogD_7.4_) descriptors (see Methods) and the quantitative structure property relationship (QSPR) model for intravitreal clearance^20^. The principal component analyses (PCA) indicated that the statins lie within the applicability domain of the quantitative structure property relationship (QSPR) model (Supplementary Table S1, Sect. 2). The rabbit CL_ivt_ values and the translation into human eye are presented in Supplementary Table S1, Sect. 3. The predicted human CL_ivt_ values of simvastatin, atorvastatin, and rosuvastatin were 2.27 mL/h, 0.67 mL/h and 0.55 mL/h, respectively. The vitreal half-lives of the statins were 1—5 h (Supplementary Table S1, Sect. 3) indicating that there would be rapid elimination from the vitreous. Thus, specialized drug delivery systems would be needed to achieve sufficient local statin concentrations in the vitreous as otherwise their action would be too short for clinical therapy.

### ARPE-19 cells tolerate statins

ARPE-19 cells were exposed to three different statins at seven different concentrations ranging from 0.5 µM to 20 µM for 24 h (Supplementary Fig. S1). According to these trial results, the concentrations of 1.5 µM, 5 µM, and 10 µM were selected for further studies. Simvastatin caused some cell membrane rupturing within 24 h but the effect had alleviated by 48 h (Supplementary Fig. S2A and S2B). Upon LPS exposure, the levels of lactate dehydrogenase (LDH) were elevated after the 24 h simvastatin exposure but nonetheless remained lower than in the cells without LPS treatment (Supplementary Fig. S2A). Collectively, LPS appeared to reduce the acute toxicity of simvastatin on cell membranes. No cellular toxicity of simvastatin was evident in the 3-(4, 5-dimethylthiazol-2-yl)-2, 5-diphenyltetrazolium bromide (MTT) test (Supplementary Fig. S2C,D) suggesting that the toxicity of simvastatin did not reach the intracellular space.

Atorvastatin was well tolerated by ARPE-19 cells with and without LPS (Supplementary Fig. S3). Only 5 µM atorvastatin in the absence of LPS reduced cell viability when determined using the MTT assay but the cell viability still remained within the 95% value when compared to its DMSO control.

In DMSO, rosuvastatin protected the integrity of the cell membrane (in comparison to the DMSO control) but for reasons unknown, the LDH levels of DMSO samples were exceptionally high (Supplementary Fig. S4A). Collectively, rosuvastatin dissolved in DMSO tended to exert acute toxic effects on the cell membrane at the 48 h time-point, but less than simvastatin (Supplementary Fig. S2A,B). In contrast to simvastatin, rosuvastatin in DMSO caused more damage to the cell membrane when the cells were exposed to LPS (Supplementary Fig. S4A) but this phenomenon was mitigated by 48 h, when rosuvastatin appeared to protect cells from LPS-induced cell membrane rupturing (Supplementary Fig. S4B). In those experiments, DMSO induced high amounts of LDH release (Supplementary Fig. S4) but by the 48 h time-point, rosuvastatin + 24 h LPS exposure returned the LDH release close to the control levels. In other words, the toxic effect of rosuvastatin in DMSO seemed to be transient. In the MTT assay, rosuvastatin was well tolerated (Supplementary Fig. S4C,D). Cell viability remained at 88% when compared to the DMSO control.

Since our in silico calculations predicted that rosuvastatin would be the least lipophilic compound of the evaluated statins, we tested its effect on cells when it was dissolved in water. Rosuvastatin dissolved in either DMSO or water exerted different effects on the cell membrane integrity since water-dissolved rosuvastatin did not cause any changes in the LDH levels within 24 h irrespective of the presence of LPS (Supplementary Fig. S5A). However, although rosuvastatin in water interfered with the cell membrane integrity at 48 h (with LPS) (Supplementary Fig. S5B), it did not compromise cell viability in the MTT assay (Supplementary Fig. S5C,D).

### Statins alleviate the LPS-induced production of pro-inflammatory cytokines in ARPE-19 cells

**Figure 1:**
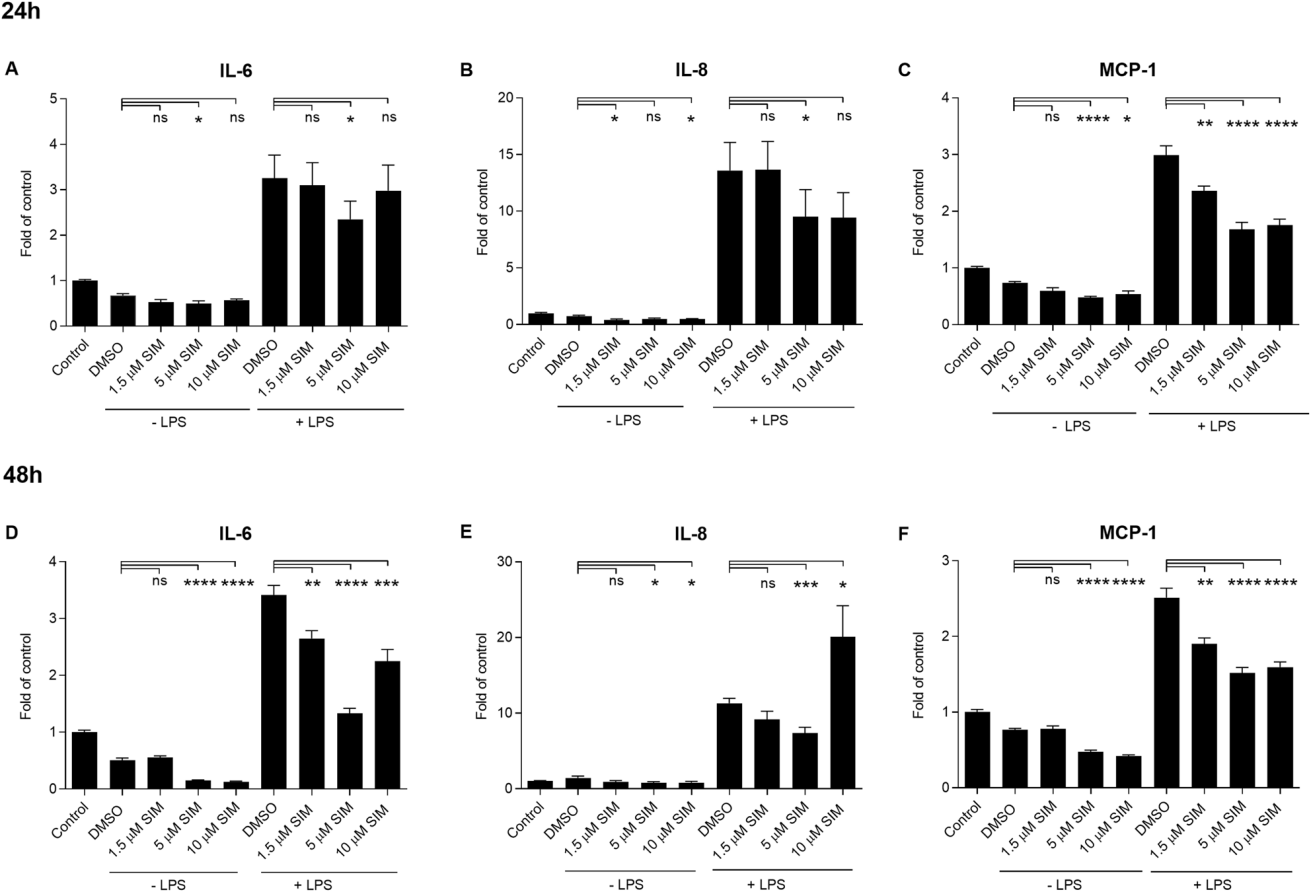
The effect of simvastatin (SIM) on the production of pro-inflammatory cytokines in human RPE cells. The levels of IL-6, IL-8, and MCP-1 were measured following the 24 h (**A**–**C**) or 48 h (**D**–**F**) exposure to SIM with or without additional exposure to LPS for 24 h. The results are represented as fold of control, which was set to be 1. Results are combined from 3 independent experiments with 4 parallel samples per group in each experiment (**A**–**C**) and from 4 independent experiments with 4 parallel samples per group in each experiment (**D**–**F**) and shown as mean ± standard error mean (SEM). *P < 0.05, **P < 0.01, ***P < 0.001, ****P < 0.0001, *ns* not significant, Mann–Whitney *U*-test.

In an attempt to clarify the anti-inflammatory properties of statins, ARPE-19 cells were exposed to different concentrations of atorvastatin, simvastatin, or rosuvastatin for 24 h or 48 h followed by a 24 h incubation with LPS, which induces the production of IL-6, IL-8, and MCP-1. Simvastatin reduced the release of IL-6 and IL-8 at the 5 µM concentration when added 24 h prior to LPS (Fig. 1A,B). A decreasing trend was visible also with 10 µM simvastatin in the production of IL-8 (Fig. 1B). All tested concentrations reduced the release of MCP-1 when simvastatin was added to cells 24 h before LPS (Fig. 1C). A longer exposure (48 h) to all concentrations of simvastatin prior to LPS reduced the release of IL-6 and MCP-1 (Fig. 1D,F), whereas only 5 µM reduced the production of IL-8 (Fig. 1E).

**Figure 2:**
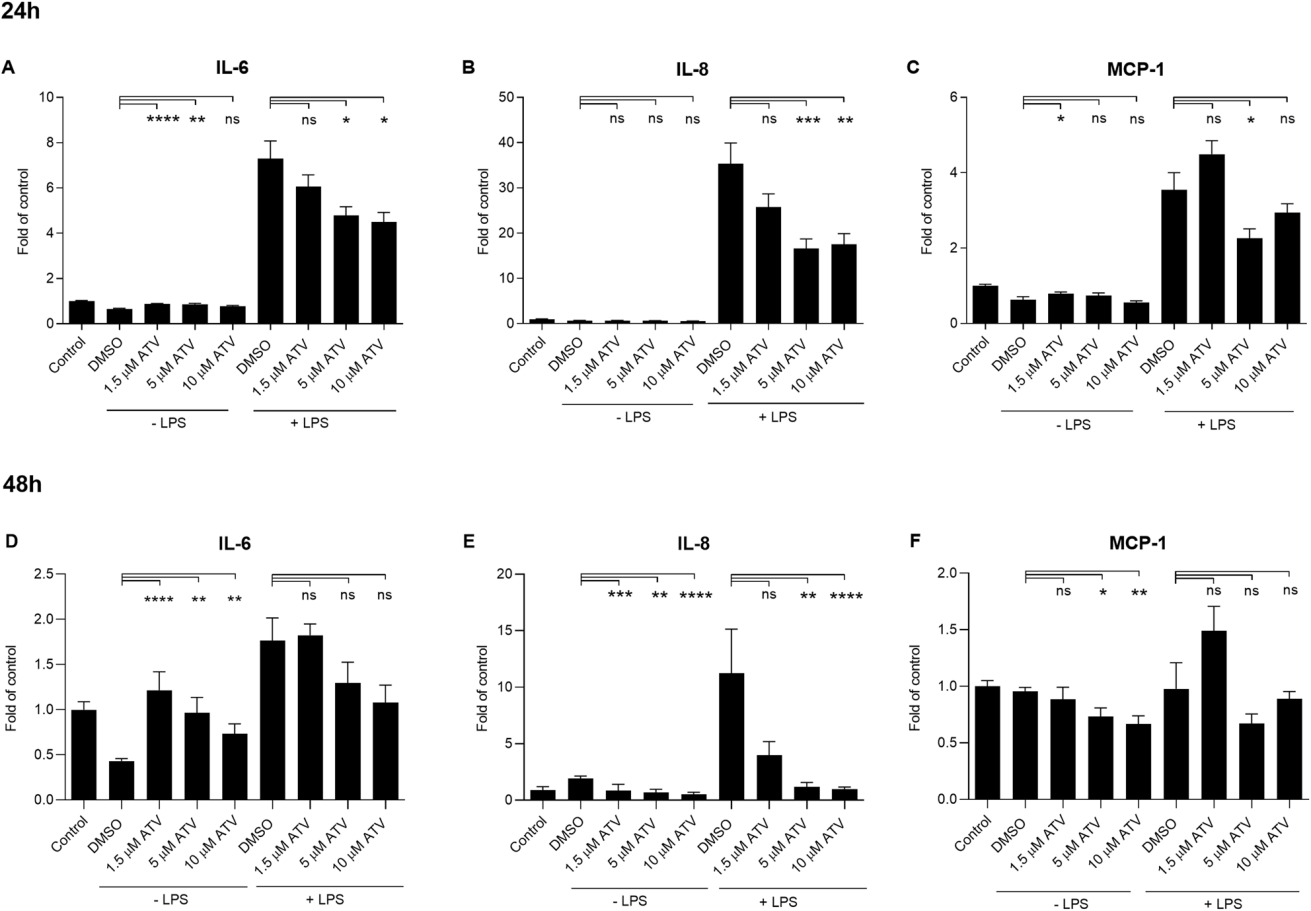
The effect of atorvastatin (ATV) on the production of pro-inflammatory cytokines in human RPE cells. The levels of IL-6, IL-8, and MCP-1 were measured following the 24 h (**A**–**C**) or 48 h (**D**–**F**) exposure to ATV with or without additional exposure to LPS for 24 h. The results are represented as fold of control, which was set to be 1. Results are combined from 4 independent experiments with 4 parallel samples per group in each experiment (**A**–**C**) and from 3 independent experiments with 4 parallel samples per group in each experiment (**D**–**F**) and shown as mean ± SEM. *P < 0.05, **P < 0.01, ***P < 0.001, ****P < 0.0001, *ns* not significant, Mann–Whitney *U*-test.

Atorvastatin pre-treatment at 5 µM or 10 µM for 24 h resulted in 1.5 and 1.6 times lower secretion of IL-6, respectively, when compared to the solvent control (Fig. 2A). In addition, 24 h exposure to 5 µM or 10 µM atorvastatin reduced the LPS-induced release of IL-8 (Fig. 2B) and MCP-1 (Fig. 2C). After the 48 h atorvastatin exposure, similar trends were seen in the cytokine production (Fig. 2D–F). In summary, atorvastatin efficiently reduced the secretion of IL-6, IL-8 and MCP-1 at 5–10 µM concentrations.

**Figure 3:**
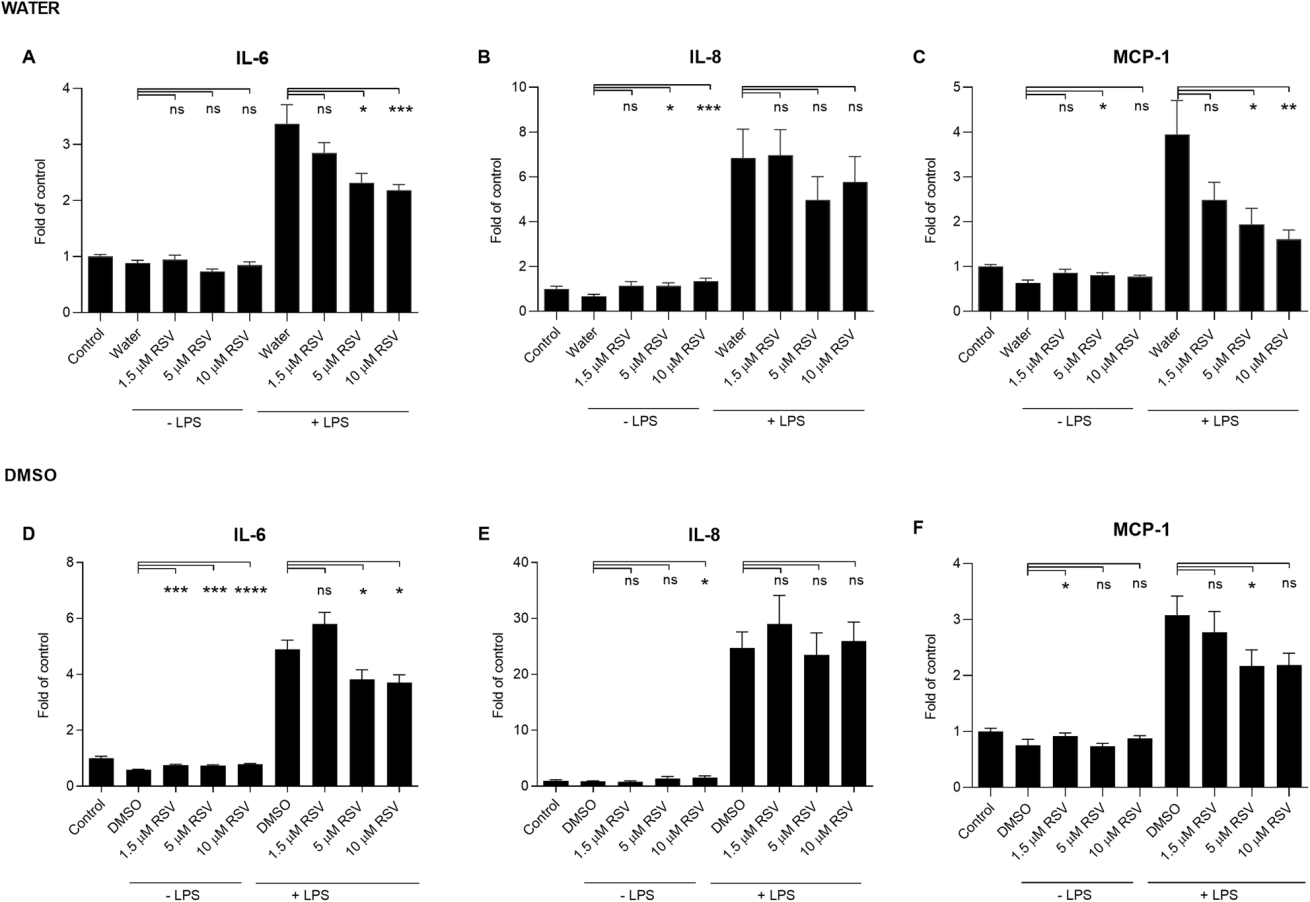
The effect of rosuvastatin (RSV) on the production of pro-inflammatory cytokines in human RPE cells at 24 h incubation. The levels of IL-6, IL-8, and MCP-1 were measured following the exposure of RSV dissolved in water (**A**–**C**) or DMSO (**D**–**F**) with or without additional exposure to LPS for 24 h. The results are represented as fold of control, which was set to be 1. Results are combined from 3 independent experiments with 4 parallel samples per group in each experiment (**A**–**C**, **F**) and from 4 independent experiments with 4 parallel samples per group in each experiment (**D**–**E**) and shown as mean ± SEM. *P < 0.05, **P < 0.01, ***P < 0.001, ****P < 0.0001, *ns* not significant, Mann–Whitney *U*-test.

**Figure 4:**
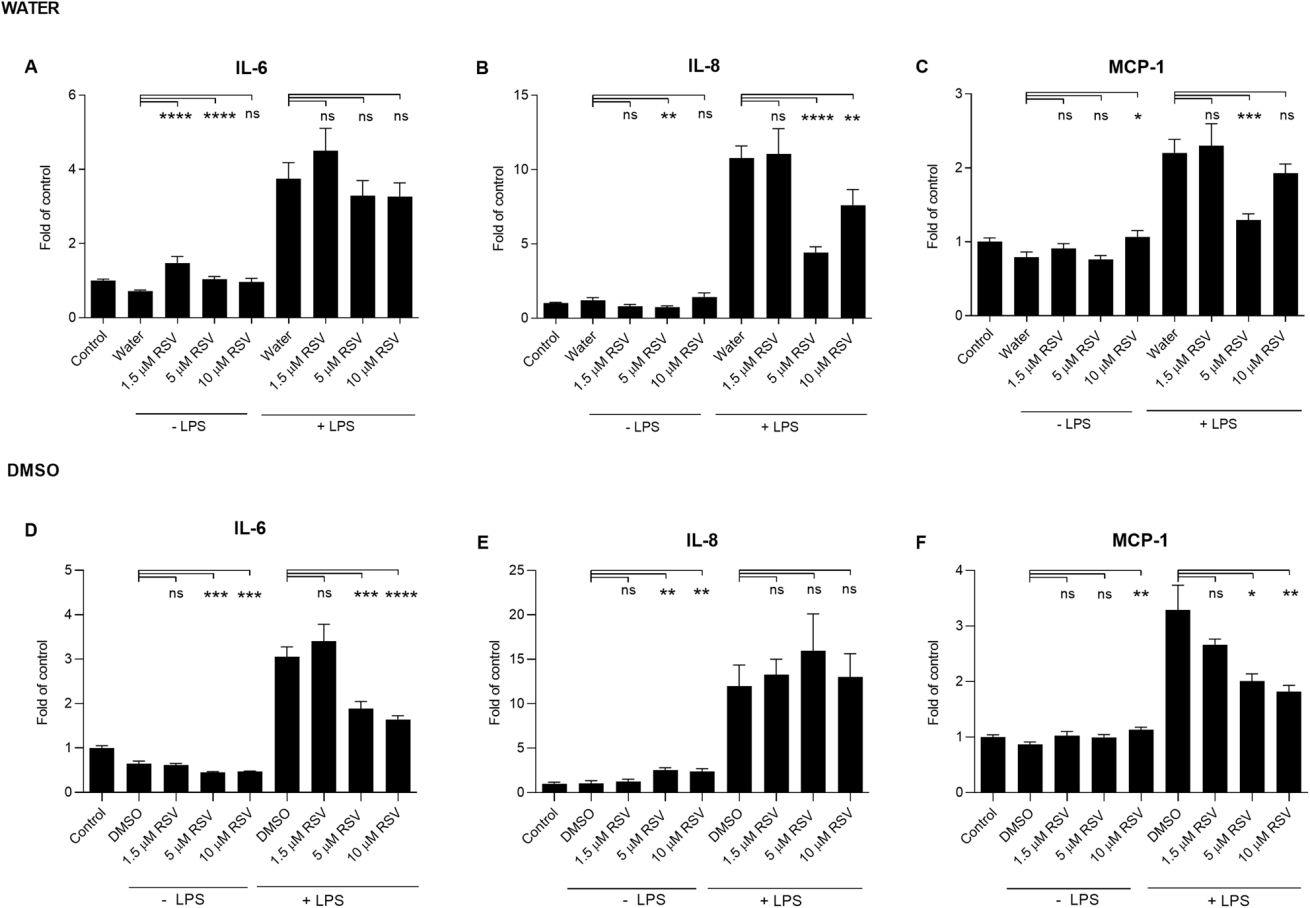
The effect of rosuvastatin (RSV) on the production of pro-inflammatory cytokines in human RPE cells at 48 h incubation. The levels of IL-6, IL-8, and MCP-1 were measured following the exposure of RSV dissolved in water (**A**–**C**) or DMSO (**D**–**F**) with or without additional exposure to LPS for 24 h. The results are represented as fold of control, which was set to be 1. Results are combined from 4 independent experiments with 4 parallel samples per group in each experiment (**A**–**C**) and from 3 independent experiments with 4 parallel samples per group in each experiment (**D**–**F**) and shown as mean ± SEM. *P < 0.05, **P < 0.01, ***P < 0.001, ****P < 0.0001, *ns* not significant, Mann–Whitney *U*-test.

The anti-inflammatory properties of rosuvastatin were tested in both DMSO and water. After the 24 h exposure, rosuvastatin resulted in similar results with both solvents; IL-6 levels were reduced after the exposure to 5 µM and 10 µM of rosuvastatin, and MCP-1 was decreased with the 5 µM concentration (Fig. 3). Likewise, IL-8 levels were reduced upon 24 h exposure to rosuvastatin at 5 µM (Fig. 3B,E). Similar decreasing trends were visible in IL-6 and MCP-1 levels after rosuvastatin exposure for 48 h, even though statistical significance was not reached in all cases (Fig. 4).

Collectively, our cell culture studies suggest that concentrations of 5 µM of simvastatin, atorvastatin, and rosuvastatin were efficient in reducing the LPS-induced release of proinflammatory cytokines IL-6, IL-8, and MCP-1 from human ARPE-19 cells.

### Vitreal drug concentrations in patients receiving oral statin therapy

The concentrations of per oral statins and their metabolites were determined in the vitreous samples of twenty patients (Supplementary Table S2). The range of the vitreal statin concentrations were 27–316 pg/mL and the metabolite levels were 39–209 pg/mL. Simvastatin (dose 40 mg daily) concentrations in the vitreous ranged from 0.027 to 0.073 ng/mL (Table 2). Vitreal drug concentrations of two patients, one with daily 10 mg simvastatin treatment and the other with daily 20 mg, were below the limit of quantification. The samples from patients treated with atorvastatin (10–40 mg daily) or rosuvastatin (10–20 mg daily) showed a concentration range from 53 to 217 pg/mL and of 52–316 pg/mL, respectively (Table 2).

**Table 2:** Pharmacokinetic parameters from single-dose administration studies of oral statins, including calculated average concentration in plasma (C_ss,av_, using Eq. 1), and the corresponding expected vitreal concentrations (Calc. Cv, using Eq. 3).

Drug	Dose (mg/d)	f_u_^a^	C_max_ (ng/mL)	AUC_0−∞_ (ng/mL)	n	Country	C_ss,av_ (ng/mL)	Ref	Calc. Cv (ng/mL)	Measured Cv (ng/mL)				
										Range		Average	SD	n
Simvastatin	40	0.05	9.8	40.32	17	China	1.68	^20^	0.084	*0.027*	*0.073*	*0.048*	*0.017*	*3*
Atorvastatin	10		3.2	11.67	24	Jordan	0.49	^21^	0.024					
		0.02	10.3	55.38	73	India	9.67	^22^	0.046			*0.104*		*1*
			17.1	117.13	24	Turkey	2.31	^23^	0.098					
	20		5.1	58.60^b,c^	12	USA	4.88	^24^	0.049					
			10.8	44.51	45	China	1.85	^25^	0.037	*0.053*	*0.217*	*0.135*	*0.116*	*2*
			15.4	183.00^b^	16	UK	7.63	^26^	0.153					
	40		6.9	98.70	18	USA	4.11	^27^	0.082					
			13.4	54.20	10	Finland	2.26	^28^	0.045			*0.207*		*1*
			12.7	61.40	12	Finland	2.56	^29^	0.051					
Rosuvastatin	10	0.12	10.8	102.59	12	China	4.27	^30^	0.513			*0.055*		*1*
			4.5	48.39	18	Germany	2.02	^31^	0.242					
	20		19.2	176.59	12	Chinese	7.36	^30^	0.883	*0.052*	*0.316*	*0.129*	*0.092*	*4*
			6.1	63.10	6	UK	2.62	^32^	0.314					

The measured C_v_ range, average, standard deviation (SD) from patients measured in the present study on the same oral statin treatment. F_u_: free fraction of drug in plasma, C_max_: maximum drug concentration in plasma, AUC_0−∞_: area under the drug concentration curve from time 0 to infinity, n: number of participants in the clinical study.

^a^From the summary of product characteristics document.

^b^The analytical method to measure the concentrations of drug in the plasma samples was based on bioassay that may be inaccurate.

^c^AUC from 0 to 24 h.

Five samples of patients without statin treatment served as negative controls. Interestingly, one of those patients presented a signal of rosuvastatin and a smaller signal of n-desmethyl rosuvastatin. This result suggests that the patient had taken a rosuvastatin tablet without reporting this to us. The other control samples did not show any traces of statins (Supplementary Table S2).

### Vitreal statin concentrations are similar with typical unbound statin concentrations in the plasma

The average steady-state concentrations (C_ss,average_) of statins in the plasma and respective unbound concentrations were calculated as described in the Methods using the data from the literature^21–33^ (Table 2). The expected drug concentrations in the human vitreous (C_v_) were estimated to be equal to the unbound drug concentrations in plasma.

**Figure 5:**
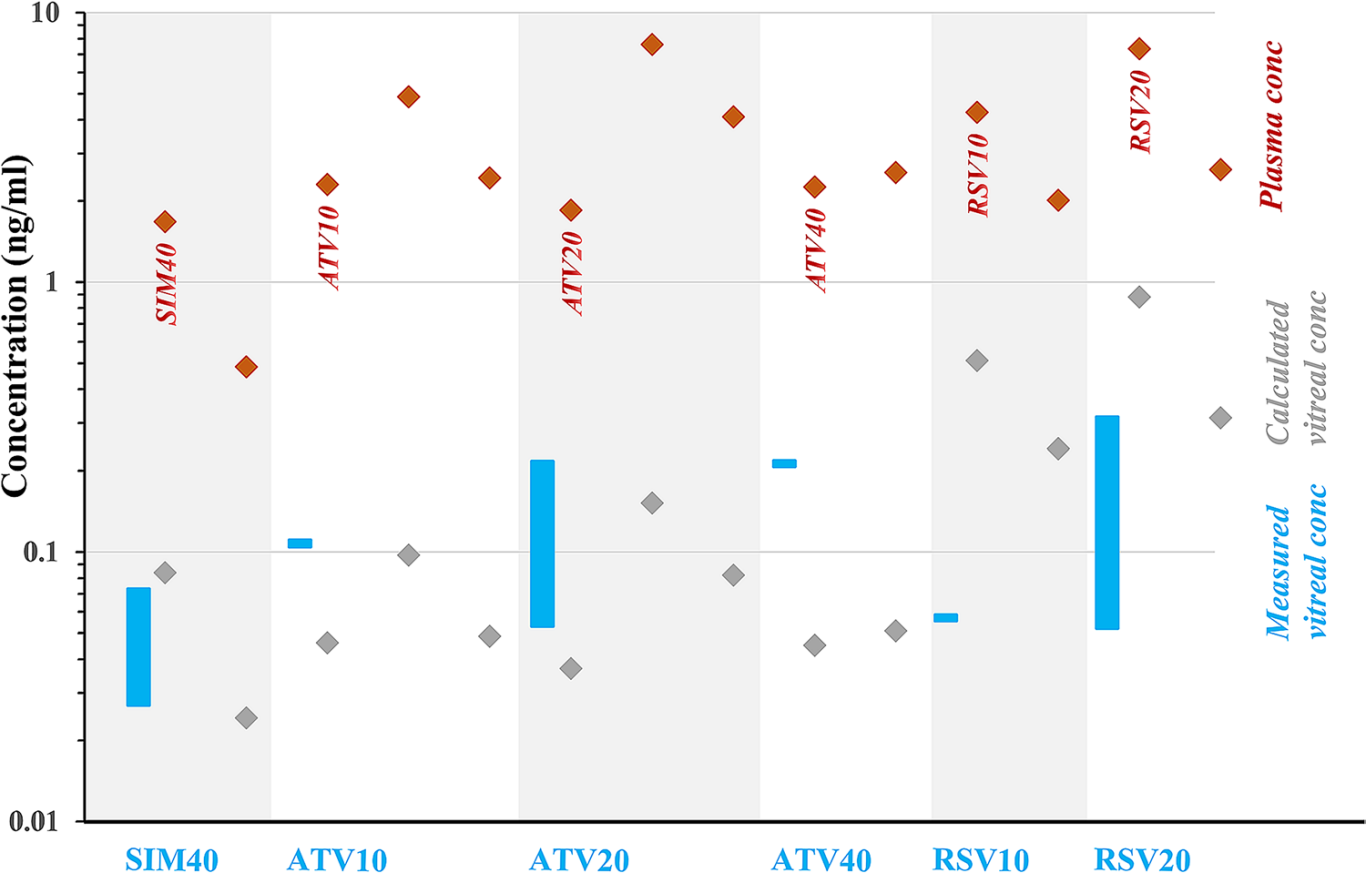
Graphical representation of the concentration of orally administered simvastatin 40 mg (SIM40), atorvastatin 10 mg (ATV10), 20 mg (ATV20), 40 mg (ATV40), and rosuvastatin 10 mg (RSV10), 20 mg (RSV20) in plasma (from the literature^22–34^, red diamonds) and in vitreous (from the present study, blue rectangles). Grey diamonds represent the calculated C_v_ based on the corresponding plasma concentration.

The comparison in Fig. 5 reveals that the statin concentrations in plasma (C_ss, average_, red diamonds) are 1–2 orders of magnitude higher than the concentrations in the vitreous (C_v_, blue rectangles). In contrast, the unbound concentrations in plasma (equivalent to calculated C_v_, grey diamonds) are within the same range with the measured statin C_v_. This indicates that only the unbound drug is capable of passing through blood-ocular barriers.

### Pharmacokinetic feasibility analysis of intravitreal statin treatment

Local delivery of statins during the RRD surgery is an interesting option to prevent PVR and other inflammatory complications, but statins dissolved in solution and administered by injection would be eliminated too rapidly to achieve the therapeutic goals (short vitreal half-lives shown in Supplementary Table S1, Sect. 3). Therefore, we simulated the performance of different intravitreal drug delivery systems for statins.

**Figure 6:**
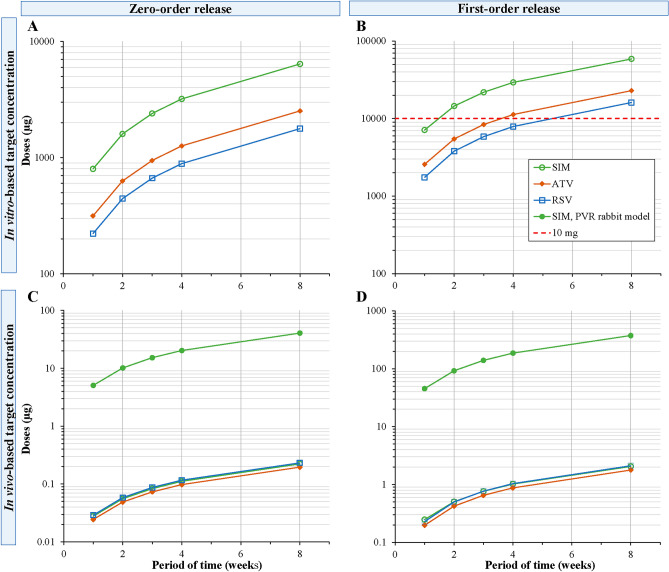
Plots presenting the doses of simvastatin (SIM), atorvastatin (ATV), and rosuvastatin (RSV) required to load in drug delivery systems to achieve target concentration based on the in vitro (**A**, **B**) or in vivo concentrations (**C**, **D**) presented in Table 3 for time treatment between one week to 2 months depending on the risk level of harmful secondary fibrosis in RRD patients. The red dashed line represents the maximum loading for 100 µl intravitreal delivery system, the filled green circles represents the doses required based on simvastatin effective concentration in PVR rabbit model (^16^, calculation shown in “Materials and Methods”).

Numerous simulations were performed to estimate the required statin doses in the delivery systems that would be needed to maintain therapeutic drug concentrations for defined periods. Three target concentrations were aimed (1) 5 µM based on the in vitro cell experiments (Fig. 6A,B), (2) the highest experimental statin concentration detected in vivo in patients (Fig. 6C,D) and (3) the calculated therapeutic concentration for simvastatin in PVR animal model^16^ (filled circles, Fig. 6C,D). Table 3 shows the required loading doses for the statins as a function of dosing interval. In the case of a 5 µM target, 1.8–6.4 mg and 16–59 mg doses would be required for two months treatment with zero-order and first-order systems, respectively. On the other hand, less than 2.2 µg would be adequate to maintain the statin concentrations at the highest levels observed in the clinical pharmacokinetic study. Whereas, less than 375 µg of simvastatin would be recommended for reaching the therapeutic concentration observed in the preclinical PVR model^16^ for a period of two months delivery.

**Table 3:** Loading doses required for the statin drug delivery systems with zero- and first-order release rate in order to achieve 5 µM concentration (2092.8 ng/mL for simvastatin, 2793.2 ng/mL for atorvastatin and 2407.7 for rosuvastatin), the highest vitreal concentration measured in the vitreous of patients (0.07 ng/mL for simvastatin, 0.22 ng/mL for atorvastatin and 0.32 ng/mL for rosuvastatin) and the calculated simvastatin therapeutic concentration in PVR animal model^16^ of 13.3 ng/mL.

	Target conc	Zero-order release Loading dose (µg)			First-order release Loading dose (µg)		
	(ng/mL)	for 1 week	for 3 weeks	for 8 weeks	for 1 week	for 3 weeks	for 8 weeks
Simvastatin	2092.8	799.6	2399	6397	7116	21,883	58,797
	13.3	5.09	15.27	40.71	45.28	139.3	374.2
	0.07	0.028	0.084	0.223	0.248	0.763	2.051
Atorvastatin	2793.2	314.9	944.7	2519	2570	8363	22,899
	0.22	0.024	0.073	0.196	0.198	0.650	1.779
Rosuvastatin	2407.7	221.9	665.7	1775	1743	5841	16,088
	0.32	0.029	0.087	0.233	0.229	0.767	2.111

## Discussion

Lipophilic statins have demonstrated effective pleiotropic (i.e. anti-inflammatory, neuroprotective) properties in RRD eyes^12,34^. The use of regular statin treatment to reduce the plasma cholesterol levels has resulted in a 28% reduced risk of re-vitrectomy in patients who were operated due to RRD^12^. In addition to this epidemiological study, also a clinical trial has suggested that statin use might be beneficial for RRD eyes^34^. We were interested in clarifying the effects of statins on RPE cells as well as determining the drug concentrations present in the eyes after systemic medication.

Simvastatin, atorvastatin and rosuvastatin were able to inhibit the LPS-induced expression of pro-inflammatory cytokines IL-6, IL-8, and MCP-1. The activity was achieved at the 5 µM statin concentrations i.e. high concentrations were needed to combat the inflammation induced by LPS, which is a much stronger stimulant than conditions associated with surgery. According to the putative anti-inflammatory potential of statins, it is likely that the anti-inflammatory effects and the prevention of PVR in vivo could be achieved at concentrations lower than 5 µM, which would be in line with the epidemiological and clinical studies^12,34^.

According to the in silico predictions of LogD_7.4_, simvastatin displayed higher lipophilicity than rosuvastatin. DMSO-dissolved rosuvastatin tended to exert some acute toxic effects on the cell membrane, whereas water-dissolved rosuvastatin showed no changes in LDH levels within 24 h with or without the influence of LPS. The main transport mechanism for crossing the blood-ocular barrier may be by transmembrane diffusion, suggesting that the lipophilic compounds are more permeable (simvastatin > atorvastatin > rosuvastatin). In addition, simvastatin may be actively transported by the monocarboxylate transporters family members present in the blood-retinal barrier^35–38^ and by some other novel organic cation transporters^39^. However, protein binding in plasma has been suggested to be an important determinant of the ocular distribution of systemic drugs^36^. Our results strongly support this concept, as the vitreous concentrations at steady state were about the same as their unbound concentrations in plasma, but 1–2 orders of magnitude lower than the total concentrations in plasma. For that reason, the most hydrophilic statin, rosuvastatin with the highest f_u_, exhibited the highest concentrations in vitreous. Even though there is variability in statin pharmacokinetics due to the racial differences, and possible polymorphisms^40–42^, these factors would not affect our results, which revealed similarities between the measured and calculated vitreal concentrations.

Intravitreal administration of statins during the vitrectomy operation would represent an attractive option as a way of improving the recovery of the operated eye after surgery by decreasing the risk of PVR formation and need for reoperations. The local administration may result in higher, and more effective, drug concentrations than could be delivered by per oral tablet therapy. This approach was used in a preclinical PVR rabbit model^16^. In that study, four injections of 15 µM simvastatin were injected intravitreally at intervals of two days. Clear beneficial effects were seen at the end point of 28 days. Since intravitreal injections to the patients are not feasible at two-day intervals, we simulated the prospects of a still-to-be devised long acting intravitreal drug delivery system. The preclinical study of Kawahara^16^ and the clinical study of Loukovaara and co-workers^12^ suggest that lower statin concentrations in vitreous could be adequate to achieve clinical effects. The estimated therapeutic vitreal concentration from Kawahara et al. preclinical study was in the range of 3 M^−8^ (13.3 ng/mL), while the vitreal concentrations of oral statins (three compounds, different doses) during patient chronic medication were 0.02–0.3 ng/mL (i.e. 0.6–7 M^−10^). Similar vitreal concentrations as in the clinical pharmacokinetic study could be maintained for 2 months with a microgram scale dose, as compared to the per oral doses of 600–2400 mg. In the case of aiming to achieve 3 M^−8^ level for the same time treatment, less than 375 µg are required for first-order release delivery system, and 40 µg for the zero-order release. Even today, these kinds of small doses of statins can be formulated with many controlled release technologies.

Our present findings support the idea that modulation of intraocular inflammation with statins has clinical potential in reducing PVR development; for example, statins could be injected during surgery into the vitreous in a specialized drug delivery system.

## Materials and methods

### Cell studies

#### Cell cultures

Human ARPE-19 cell line was purchased from the American Type Culture Collection (ATCC; VA, USA). The cells were grown on 10 cm culture plates in a humidified 5% CO_2_ atmosphere at 37 °C in Dulbecco’s Modified Eagle’s Medium (DMEM) and nutrient F-12 1:1 mixture (Life Technologies, USA) containing 10% Fetal Bovine Serum (FBS; Thermo Fisher Scientific, USA), 100 mg/mL penicillin, 100 µg/mL streptomycin, and 2 mM l-glutamine (Lonza, Switzerland). In the experiments, the cells were split into 12-well culture plates at 200,000 cells/mL/well in serum-containing medium for 55 h before drug exposure. Confluent cell cultures were washed with serum-free medium and the cells were treated with 1.5 µM, 5 µM, and 10 µM concentrations of atorvastatin, simvastatin, or rosuvastatin (all from Sigma Aldrich, USA) for 24 h or 48 h. After statin incubation, 10 µg/mL lipopolysaccharide (LPS; Sigma Aldrich, USA; cat no L 6529) was added for an additional 24 h.

#### Preparation of samples

Medium samples and cell lysates were collected after the LPS exposure. The cells were washed with Dulbecco’s Phosphate Buffer Saline (DPBS; Lonza) prior to adding 50 µl/well of the Mammalian Protein Extraction Reagent (M-Per; Thermo Scientific, cat. #78501). Media samples were centrifuged at 380 g for 10 min, transferred into clean tubes and stored at − 20 °C prior to the analyses of IL-6, IL-8, and MCP-1. The cells were collected by scraping and were centrifuged at 16,060×*g* for 20 min. The lysates for Enzyme-Linked Immunosorbent Assay (ELISA) assays were transferred into clean tubes and stored at − 70 °C until analysed.

#### Cell viability assays

Cell viability was assessed by determining the amount of LDH enzyme from culture media using a commercial kit (Promega Corporation, USA) according to the manufacturer’s protocol. This assay provides information about the integrity of the plasma membranes of the cells (high LDH levels meaning cell damage). In addition, the cell viability was examined with MTT assay, as described previously^43^. In viable cells, the mitochondrial succinate dehydrogenase enzyme can metabolize the MTT dye into formazan that absorbs light at the 550 nm wavelength.

#### Enzyme-linked immunosorbent assay

Pro-inflammatory cytokines IL-6, IL-8, and MCP-1 were determined using a specific ELISA from BD Pharmingen, San Jose, CA, USA according to the manufacturer’s protocols.

#### Statistical analyses

Statistical analyses of cell experiments were conducted using the Graph Pad Prism (Graph Pad Software, San Diego, CA). Differences between the groups were analysed using the Mann–Whitney *U*-test and *P* values ≤ 0.05 were considered statistically significant. Results are shown as mean ± SEM.

### Clinical pharmacokinetic studies

#### Study design

The study was conducted according to the tenets of the Declaration of Helsinki, and was approved by the Institutional Review Board and Ethical committee of Helsinki University Central Hospital. Twenty patients were enrolled; fifteen with per oral statin treatment and five controls. Signed informed consent was obtained from each participant prior to the vitreous sampling. Confidentiality of the patient records was maintained when the clinical data were entered into a computer-based standardized data entry for analysis. Sixteen patients were being operated due to PDR; of these, five patients had also Tractional Retinal Detachment (TRD); two patients were operated due to RRD, one due to age-related epiretinal fibrosis (pucker), and one due to a macular hole (Supplementary Table S2). The vitrectomised patients on statin therapy used three different systemic statins, and eight different daily statin dosing: simvastatin 10 mg (n = 1), 20 mg (n = 1), and 40 mg (n = 3); atorvastatin 10 mg (n = 1), 20 mg (n = 3), and 40 mg (n = 1); rosuvastatin 10 mg (n = 1) and 20 mg (n = 4).

All vitrectomies were performed by the recruiting vitreoretinal surgeon (SL). Undiluted vitreous samples (up to 1000 μl) were collected at the start of the transscleral 3‐port pars plana vitrectomy (23G or 25G Alcon Instruments, Inc., Alcon Constellation Vision system, USA) without an infusion of artificial fluid. The samples were collected by manual aspiration into a syringe via the vitrectomy with the cutting function activated. Sample aliquots were transferred into sterile 1.5 mL microcentrifuge tubes (Freemont, CA, USA) and immediately frozen and stored at − 70 °C until laboratory analysis.

#### Determination of statin concentrations from patients’ vitreous

Twenty human vitreous samples were obtained from Helsinki University Central Hospital for the analyses of simvastatin, atorvastatin, rosuvastatin and some of their metabolites. All reference compounds and internal standards were purchased from Toronto Research Chemicals (Toronto, Canada). Samples (100 µl) were divided into two aliquots and kept at − 80 °C prior to analysis. In the sample pre-treatment procedure, ice-cold vitreous samples were first homogenized with Tissue Lyzer II (Qiagen, Hilden, Germany) for 45 s at 30 Hz. Homogenizer blocks were pre-cooled by placing them at − 20 °C. The homogenates (40 µl) were diluted with 120 µl of chilled acetonitrile (Chromasolv, Honeywell, Seelze, Germany) including 0.1% formic acid (Merck, Darmstadt, Germany) and 0.5 ng/mL internal standard mixture to precipitate proteins. The dilutions were first vortexed for 5 s and then centrifuged (Sigma, Osterode am Harz, Germany) at 14,400×*g* for 10 min. Supernatants (120 µl) were pipetted into analytical vials and the concentrations of the statins were measured with Ultra-high Pressure Liquid Chromatography—tandem Mass Spectrometry (UPLC-MS/MS) technique.

The same pre-treatment protocol was used to prepare calibration curves and quality control samples but homogenized and filtered porcine vitreous were used as a matrix instead of human vitreous. A minimum of six calibration curve concentrations was used for quantitation of the compounds. Two parallel calibration curve sets included all eleven analytes and four deuterated internal standards. Three quality control samples in three parallel sets were used to validate the method. The linear range varied from approximately 0.004 to 10 ng/mL depending on the analyte. Limit of quantitation was set to the smallest calibration curve point of each drug. Each sample was analysed twice and the average of these values was designated as the concentration of the sample.

For simvastatin (simvastatin lactone) one metabolite, simvastatin acid, was analysed while simvastatin lactone D6 and simvastatin acid D6 were used as an internal standard for these statins, respectively. For atorvastatin, five different metabolites were analysed: atorvastatin lactone, 2-OH atorvastatin, 4-OH atorvastatin, 2-OH atorvastatin lactone and 4-OH atorvastatin lactone. Atorvastatin D5 was used as an internal standard for all atorvastatin compounds. For rosuvastatin, two metabolites were analysed: rosuvastatin lactone and N-desmethyl rosuvastatin lactone. Rosuvastatin D6 was used as an internal standard. The parent drug, its metabolites and internal standard were analysed from the same sample in each case.

Liquid chromatography separation was carried out using a Waters Acquity UPLC instrument (Waters, MA, USA) coupled with Waters HSS T3 C18 (2.1 × 100 mm, 1.8 µm) column at 40 °C. The mobile phase consisted of 0.1% formic acid in ultrapure water (A) and 100% of LC–MS grade acetonitrile (B). The gradient elution started with 20% of B at 0–0.5 min, continuing with 20—100% B at 0.5–4.3 min. The total run time was 7.5 min including the flush and equilibrium of the column. The flow rate was set to 0.3 mL/min and injection volume 1.5 µl. After every three samples, a solvent blank was run to control for any carry over.

Mass spectrometry measurements were carried out using a Waters Xevo Triple Quadruple mass spectrometer (TQ-S) equipped with an ESI source. Positive ionization mode was used for all other statins except simvastatin acid and its internal standard simvastatin acid D6 which required the negative ionization mode. The source parameters were as follows: Capillary voltage 2.8 kV, source temperature 150 °C and desolvation temperature 500 °C. Nitrogen (Aga, Helsinki, Finland) was used as a desolvation gas (800 l/h), a cone gas (150 l/h) and a nebulizer gas (7 bar). Argon (Aga, Helsinki, Finland) was used as a collision gas (0.15 mL/min). The Multiple Reaction Monitoring (MRM) mode was used for quantification. Precursor and fragment ions, as well as other optimized parameters are listed in Supplementary Table S3. The resulting data were analysed with Waters MassLynx software V4.1.

#### Pharmacokinetic analyses of per orally administered statins

The concentrations of statins and their metabolites in the vitreous were analysed (see above) and expressed as ng/mL concentrations. These concentrations were compared to published statin concentrations in plasma. A literature search was carried out on pharmacokinetic studies after single-dose oral administration of simvastatin (40 mg), atorvastatin (10, 20 and 40 mg) and rosuvastatin (10 and 20 mg) in healthy volunteers. Maximum concentrations in plasma and the area under the concentration vs time curve values were collected. The expected average statin concentrations in plasma during multiple dose treatment were calculated for the doses that the patients received in our study (Eq. 1):

1 $${\mathrm{C}}_{\mathrm{ss,av}}={\mathrm{AUC}}_{\mathrm{single dose}}/\uptau$$

where τ is the time interval between doses. The drug concentrations in plasma (C, C_max,_ C_ss, av_) include both unbound (C_u_) and protein-bound drug. The values for protein binding were obtained from the summary of drug product characteristics for each statin. The free fraction of statin in plasma was calculated based on Eq. (2):

2 $${\text{f}}_{\text{u}}={{\text{C}}_{\text{u}}}\!\left/ \! {\text{C}}\right.$$

The expected drug concentrations in the patients’ vitreous samples were calculated according to Eq. (3)

3 $${\mathrm{C}}_{\mathrm{v}}= {\mathrm{f}}_{\mathrm{u}}\times {\mathrm{C}}_{\mathrm{ss},\mathrm{av}}$$

Equation (3) assumes that the vitreal drug concentrations equilibrate with the unbound drug concentrations in plasma, because only free drug is expected to cross the blood-ocular barriers.

### In silico studies

#### Calculation of molecular descriptors of simvastatin, atorvastatin and rosuvastatin

Chemical structures of simvastatin, atorvastatin and rosuvastatin were obtained either from PubChem (the structure-data file format from pubchem.ncbi.nlm.nih.gov) or from ACD/Dictionary (ACDlabs software, version 12, Advanced Chemistry Development, Inc., Toronto, Canada). Twenty-six ACDlabs molecular descriptors were generated (for complete list see Supplementary Table S1, Sect. 1).

#### Predicted pharmacokinetic parameters of intravitreal statins

The concentration profiles of intravitreal drugs are defined by the dose, volume of drug distribution and clearance. Based on our previous analyses, the distribution volumes (V_ss,ivt_) of statins (and other drugs) are expected to be nearly equal with the anatomical volume of human vitreous (4 mL)^20^. The CL_ivt_ of simvastatin, atorvastatin and rosuvastatin can be calculated using the QSPR model^20^ that is based on comprehensive rabbit data from intravitreal pharmacokinetic studies.

The CL_ivt_ of statins in rabbits was calculated using the QSPR model, Eq. (4):

4 $${\mathrm{LogCL}}_{\mathrm{ivt},\mathrm{Rabbit}}= -0.25269-0.53747 \left(\mathrm{LogHD}\right)+0.05189 ({\mathrm{LogD}}_{7.4})$$

Computational values of HD and LogD_7.4_ were obtained for simvastatin, atorvastatin and rosuvastatin as described in the previous section. The rabbit clearance values can be reliably scaled up to humans with the rationale described in Supplementary Table S1.

#### Simulation of intravitreal statin concentrations during controlled drug delivery

Predicted concentration profiles of the statins were simulated after administration of an intravitreal controlled release system. The simulations were performed using Stella Professional, Modelling & Simulation software (version 1.5.1, isee systems, Inc., Lebanon, USA). Values of CL_ivt_ and V_ss,ivt_ were obtained for each drug (see above) and incorporated into a one-compartmental model. In the simulations, a fourth order Runge–Kutta algorithm and 10^–5^ day time intervals were used. Two drug release types were investigated (1) constant zero-order release and (2) decreasing first-order release rate. Different target concentrations were investigated: (1) the effective concentrations from the cellular studies, (2) the highest concentrations in patients’ vitreous, (3) the therapeutic C_ss, average_ of simvastatin achieved in PVR rabbit model^16^. The C_ss, average_ obtained from Kawahara et al. study^16^ (13.3 ng/mL or 3 M^−8^) was calculated using the Eq. (5):

5 $${\text{C}}_{{{\text{ss}},{\text{ average}}}} = {\text{ dose}}/({\text{CL}}_{{{\text{ivt}}}} \times {\text{t}})$$

where the dose was 0.63 µg (0.1 mL of 15 µM simvastatin solution), τ or dosing interval was 48 h, and the rabbit CL_ivt_ of simvastatin was 0.982 mL/h (Table S1, Sect. 3).

We simulated the drug loading doses that would be needed to maintain the target concentrations in the human vitreous from one to eight weeks.

## Supplementary Information

Supplementary Information.

## Data Availability

All data generated or analysed during this study are included in this published article (and its Supplementary Information files). In addition, all data pertaining to the current study is available from the corresponding author on reasonable request.

## Abbreviations

SIM: Simvastatin
ATV: Atorvastatin
RSV: Rosuvastatin
AUC: Area under the concentration vs time curve
CL_ivt_: Intravitreal clearance
C_max_: Maximum drug concentration
C_ss, average_: Average steady-state concentrations
C_v_: Drug concentrations in the human vitreous
DMSO: Dimethyl sulfoxide
ECM: Extracellular matrix
ELISA: Enzyme-linked immunosorbent assay
fu: Free fraction of drug in plasma
HD: Hydrogen bond donor
IL: Interleukin
LDH: Lactate dehydrogenase
LogD_7.4_: Logarithm of octanol–water distribution coefficient at pH 7.4
LPS: Lipopolysaccharide
MCP-1: Monocyte chemoattractant protein-1
MMP: Matrix metalloproteinase
MTT: 3-(4, 5-Dimethylthiazol-2-yl)-2, 5-diphenyltetrazolium bromide
PCA: Principal component analyses
PDR: Proliferative diabetic retinopathy
PVR: Proliferative vitreoretinopathy
QSPR: Quantitative structure property relationship
RPE: Retinal pigment epithelial
RRD: Rhegmatogenous retinal detachment
SEM: Standard error of the mean
τ: Dosing interval
TGF-β1: Transforming growth factor-β1
TRD: Tractional retinal detachment
UPLC-MS/MS: Ultra-high pressure liquid chromatography—tandem mass spectrometry
VEGF: Vascular endothelial growth factor
V_ss, ivt_: Intravitreal volume of the distribution
