# Author Correction: Statins for the prevention of proliferative vitreoretinopathy: cellular responses in cultured cells and clinical statin concentrations in the vitreous

**DOI:** 10.1038/s41598-021-92615-z

**Published:** 2021-07-22

**Authors:** Yashavanthi Mysore, Eva M. del Amo, Sirpa Loukovaara, Marja Hagström, Arto Urtti, Anu Kauppinen

**Affiliations:** 1 School of Pharmacy, Faculty of Health Sciences, University of Eastern Finland, Yliopistonranta 1C, P.O.B. 1627, 70211 Kuopio, Finland; 2 Department of Ophthalmology, Unit of Vitreoretinal Surgery, Helsinki University Central Hospital, and Individualized Drug Therapy Research Program, University of Helsinki, Helsinki, Finland; 3 School of Pharmacy, University of Helsinki, Helsinki, Finland; 4 Institute of Chemistry, St. Petersburg State University, Petergof, Russian Federation

Correction to: *Scientific Reports* 10.1038/s41598-020-80127-1, published online 13 January 2021

This Article contains an error in Table 2, where in the column heading “AUC_0−∞_(ng/mL)” the unit “ng/mL” should read “h x ng/mL”.

In addition, in the Materials and methods section under subheading ‘Pharmacokinetic analyses of per orally administered statins’.

“The expected average statin concentrations in plasma during multiple dose treatment were calculated for the doses that the patients received in our study (Eq. 1):

$$ {\text{C}}_{{{\text{ss,av}}}}  = {\text{AUC}}_{{{\text{singledose}}}} /\tau $$

where τ is the time interval between doses.”

should read:

“The expected average statin concentrations in plasma during multiple dose treatment were calculated for the doses that the patients received in our study (Eq. 1):

$$ {\text{C}}_{{{\text{ss,av}}}} ({\text{ng}}/{\text{mL}}) = {\text{AUC}}_{{{\text{singledose}}}} ({\text{h}} \times {\text{ng}}/{\text{mL}})/\tau (24 {\text{h}}) $$

where τ is the time interval between doses that correspond to 24 h.”

