# Supplementary material for: Statins for the prevention of proliferative vitreoretinopathy: cellular responses in cultured cells and clinical statin concentrations in the vitreous
Source: Sci Rep. 2021 Jan 13;11:980. doi: 10.1038/s41598-020-80127-1 (PMC7806714; doi:10.1038/s41598-020-80127-1)
Supplement: Supplementary file 1 — Supplementary Information. [file 41598_2020_80127_MOESM1_ESM.pdf]

Statins for the prevention of proliferative vitreoretinopathy: cellular responses in cultured cells and clinical statin concentrations in the vitreous

Yashavanthi Mysore[1#*], Eva M del Amo[1#], Sirpa Loukovaara[2], Marja Hagström[3], Arto Urtti[1,3,4], Anu Kauppinen[1]

[#]Equal contribution

[*]Corresponding author

Supplementary data

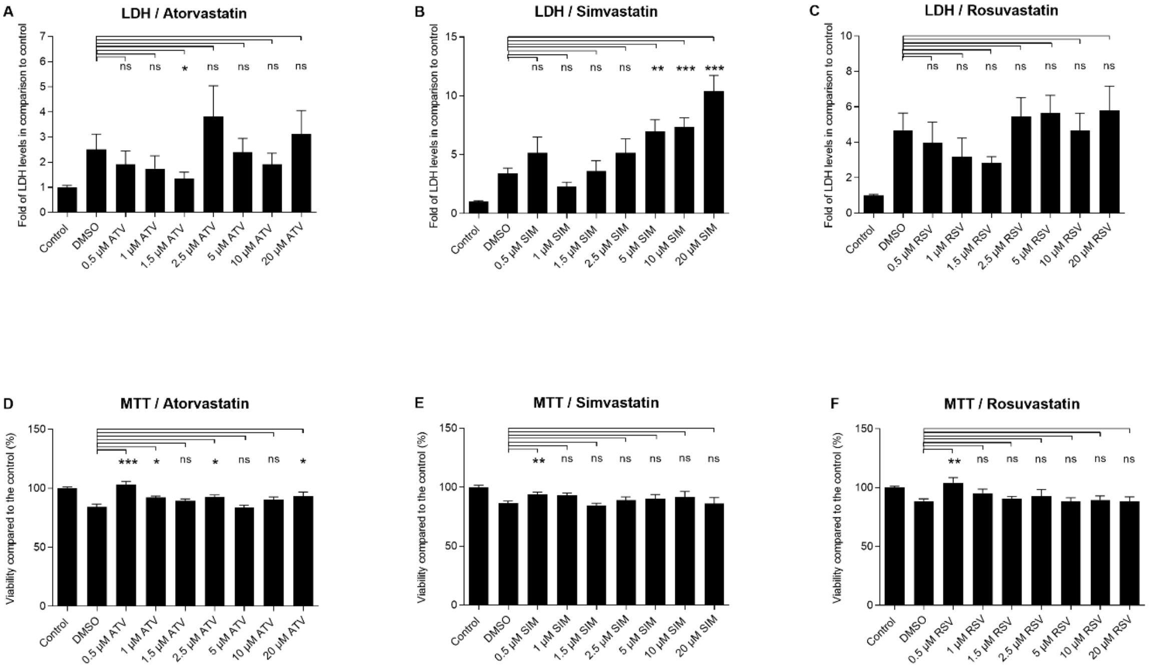

Supplementary Figure S1. Cell viability of ARPE-19 cells upon atorvastatin (ATV), simvastatin (SIM) and rosuvastatin (RSV) exposure (24 h) without lipopolysaccharide (LPS). The viability was tested with 0.5 µM, 1 µM, 1.5 µM, 2.5 µM, 5 µM, 10 µM and 20 µM ATV, SIM and RSV and represented as fold of LDH levels in comparison to control, which was set to be 1 (**A-C**) and viability represented in comparison to control, set to be 100 (%) in case of MTT (**D-F**). Results are combined from 3 independent experiments with 4 parallel samples per group in each experiment and shown as mean ± SEM. * $P < 0.05$, ** $P < 0.01$, *** $P < 0.001$, **** $P < 0.0001$, ns - not significant, Mann-Whitney U-test.

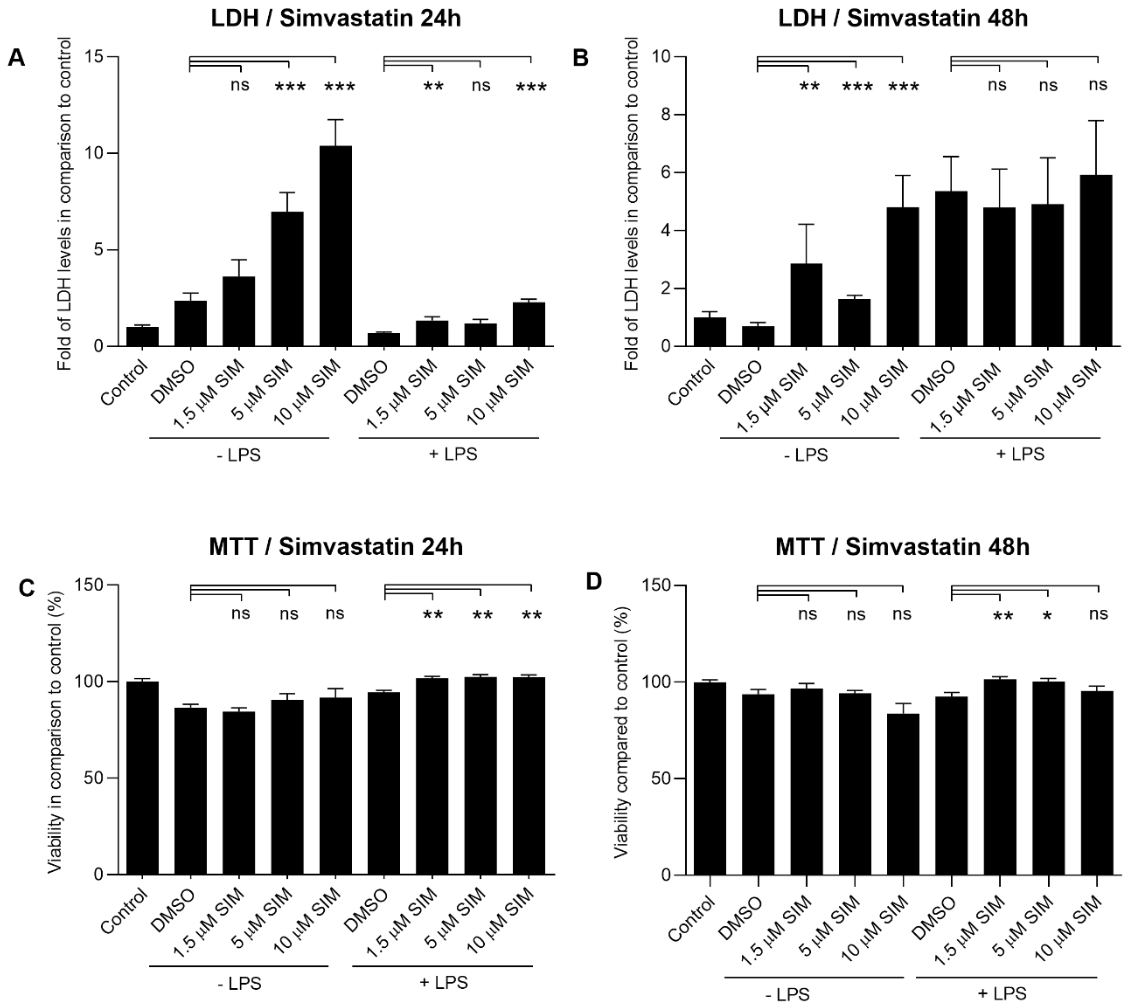

Supplementary Figure S2. Cell viability of ARPE-19 cells upon simvastatin (SIM) exposure (24h or 48h) with or without lipopolysaccharide (LPS). The viability was represented as fold of LDH levels in comparison to control, which was set to be 1 for untreated control cells (**A and B**) and viability represented in comparison to control, set to be 100 (%) in case of MTT (**C and D**). Other groups are compared individually to DMSO or DMSO with LPS. Results are combined from 2 - 3 independent experiments with 4 parallel samples per group in each experiment and shown as mean ± SEM. * $P < 0.05$, ** $P < 0.01$, *** $P < 0.001$, **** $P < 0.0001$, ns - not significant, Mann-Whitney U-test.

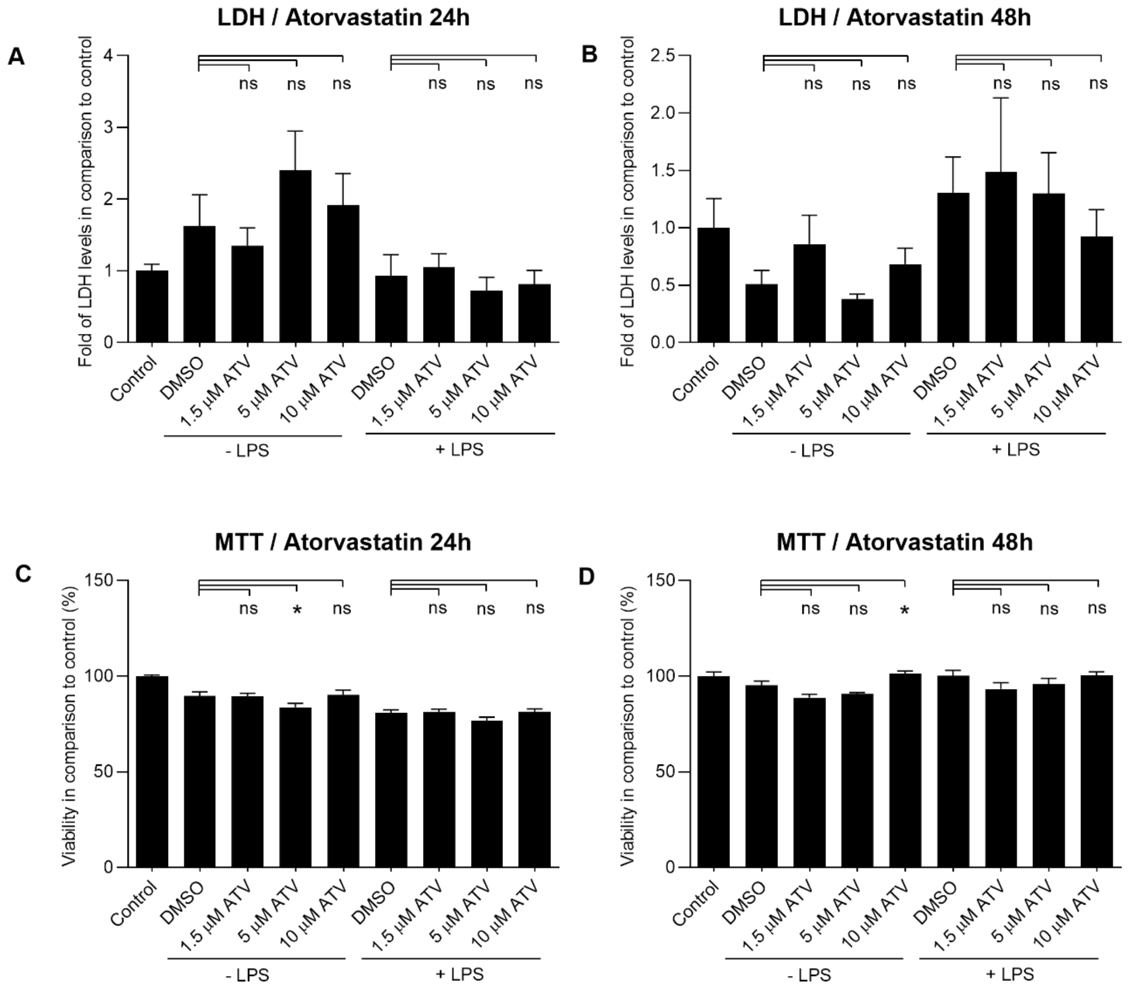

Supplementary Figure S3. Cell viability of ARPE-19 cells upon atorvastatin (ATV) exposure (24h or 48h) with or without lipopolysaccharide (LPS). The viability was represented as fold of LDH levels in comparison to control, which was set to be 1 for untreated control cells (**A and B**) and viability represented in comparison to control, set to be 100 (%) in case of MTT (**C and D**). Other groups are compared individually to DMSO or DMSO with LPS. Results are combined from 2 - 3 independent experiments with 4 parallel samples per group in each experiment and shown as mean ± SEM. * $P < 0.05$, ** $P < 0.01$, *** $P < 0.001$, **** $P < 0.0001$, ns - not significant, Mann-Whitney U-test.

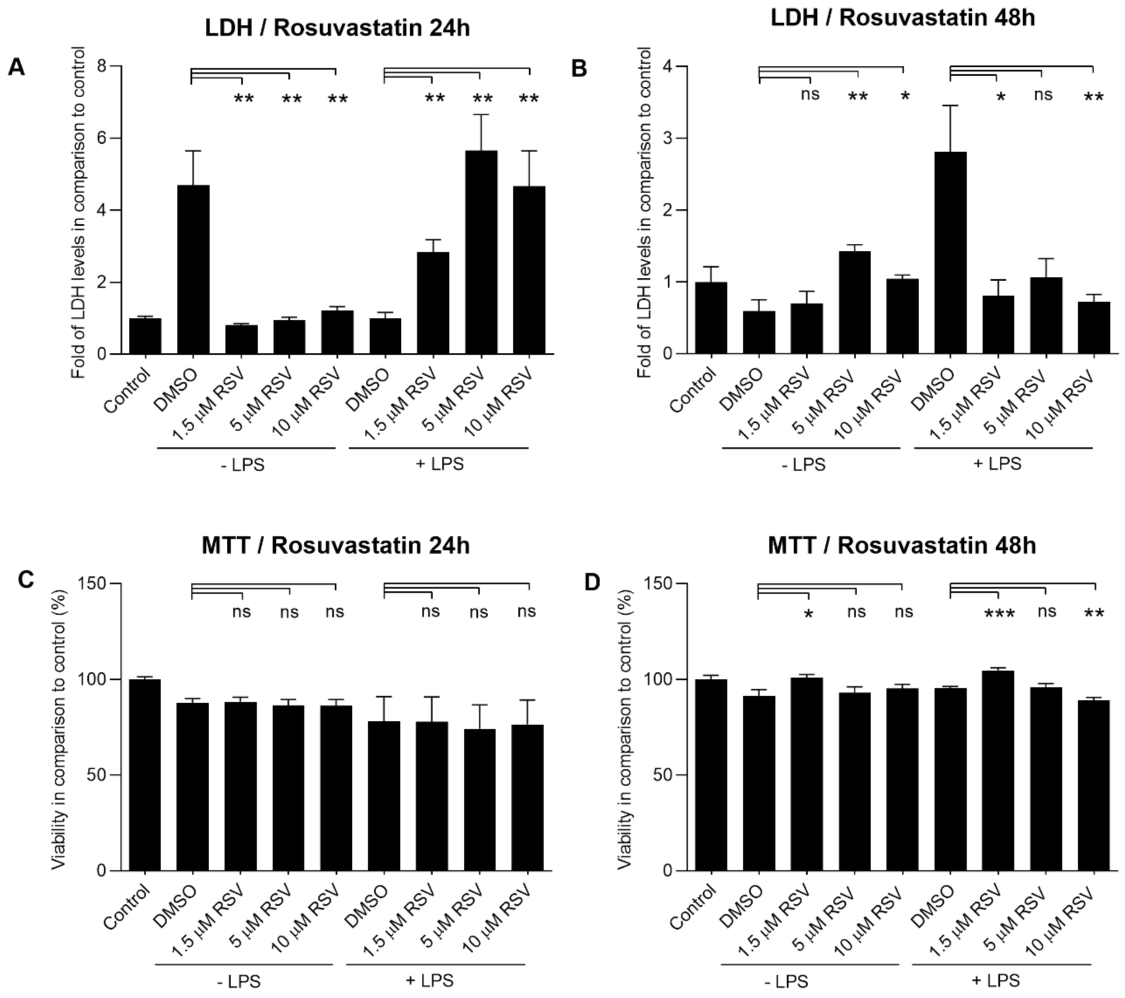

Supplementary Figure S4. Cell viability of ARPE-19 cells upon rosuvastatin (RSV) exposure (24h or 48h) dissolved in DMSO with or without lipopolysaccharide (LPS). The viability was represented as fold of LDH levels in comparison to control, which was set to be 1 for untreated control cells (**A and B**) and viability represented in comparison to control, set to be 100 (%) in case of MTT (**C and D**). Other groups are compared individually to DMSO or DMSO with LPS. Results are combined from 2 - 3 independent experiments with 4 parallel samples per group in each experiment and shown as mean ± SEM. * $P < 0.05$, ** $P < 0.01$, *** $P < 0.001$, **** $P < 0.0001$, ns - not significant, Mann-Whitney U-test.

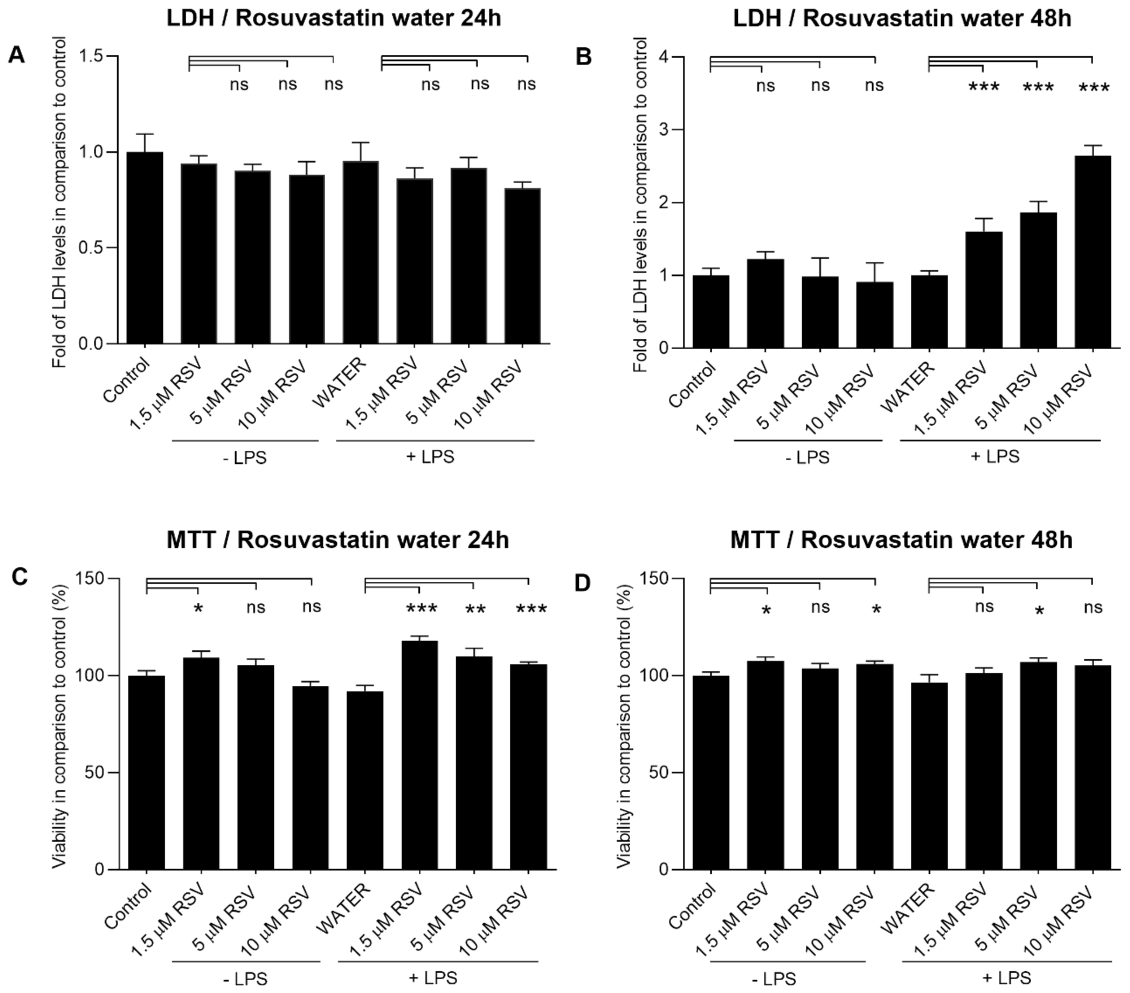

Supplementary Figure S5. Cell viability of ARPE-19 cells upon rosuvastatin (RSV) exposure (24h or 48h) dissolved in molecular grade distilled water with or without lipopolysaccharide (LPS). The viability was represented as fold of LDH levels in comparison to control, which was set to be 1 for untreated control cells (**A and B**) and viability represented in comparison to control, set to be 100 (%) in case of MTT (**C and D**). Other groups are compared individually to untreated control or water with LPS. Results are combined from 2 independent experiments with 4 parallel samples per group in each experiment and shown as mean ± SEM. * P < 0.05, ** P < 0.01, *** P < 0.001, **** P < 0.0001, ns - not significant, Mann-Whitney U-test.

1. Calculation of 26 molecular descriptors using ACDlabs software (version 12, Advanced Chemistry Development, Inc., Toronto, Canada):

From the chemical structures of simvastatin, atorvastatin and rosuvastatin (obtained from the structure-data file from PubChem or from ACD/Dictionary) the following molecular descriptors were generated: pKa for the most acidic molecular form, pKa for the most basic form, LogD at pH 5.5 and 7.4, LogP, MW, PSA (polar surface area), FRB (freely rotatable bonds), HD (hydrogen bond donors), HA (hydrogen bond acceptors), rule of 5, molar refractivity, molar volume, parachor, index of refraction, surface tension, density, polarizability, C ratio, N ratio, NO ratio, hetero ratio, halogen ratio, number of rings and number of aromatic, 4-, 5- and 6-membered rings.

2. Rabbit QSPR intravitreal clearance prediction and applicability domain

The rabbit intravitreal clearance (CL_{ivt}) model [1]:

$LogCL_{ivt,Rabbit} = -\ 0.25269–0.53747\ (LogHD) + 0.05189\ (LogD_{7.4})$

was built based on a molecule set with a defined 26 descriptor-value range. The statins should fall within this same chemical space in other to get reliable predictions for their intravitreal clearance. This can be visualised in the principal component analyses score plot (below) of the molecule set (green dots) including the statin (red dot). The ellipse indicates the applicability domain of the model. The statins lie inside the ellipse.

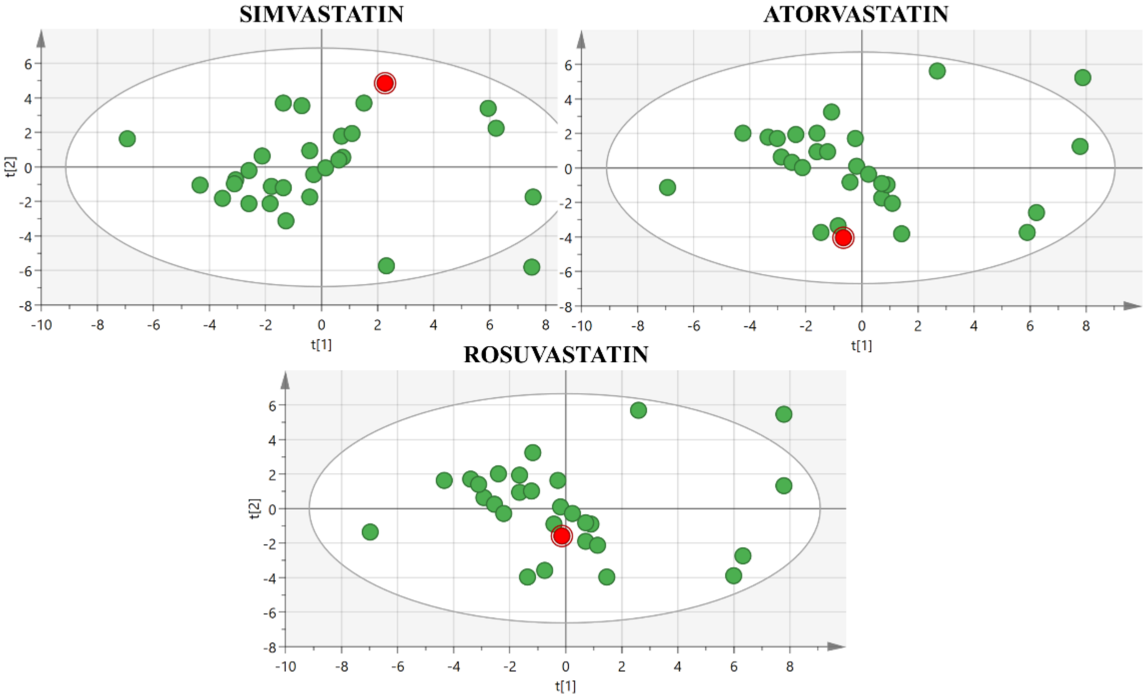

The statins presented only one to two descriptors out of the range (LogP and Log $D_{7.4}$ for simvastatin, LogP for atorvastatin, and pKa for the most basic form for rosuvastatin).

3. Scaling from rabbit intravitreal clearance to human eye rationale

Small lipophilic compounds are cleared from the vitreous mainly through the RPE [1]. The RPE surface area of rabbit eyes is 520 mm^2 [2] and 1204 mm^2 in human [3]. Based on the equation CL=P * S, where P is the drug permeability in the RPE and S is the surface area of the RPE, and assuming similar permeability of rabbit and human RPE, the human CL_{ivt} is calculated.

Human half-life ($t_{1/2,ivt}$) is calculated using the equation $t_{1/2} = (\ln 2^* V_{ss,ivt})/CL_{ivt}$ and assuming a $V_{ss,ivt}$ equal to the anatomical human vitreous (4 ml).

	Rabbit predicted CL_{ivt} (ml/h)	Human predicted CL_{ivt} (ml/h)	Human $t_{1/2,ivt}$ (h)
Simvastatin	0.982	2.274	1.2
Atorvastatin	0.290	0.671	4.1
Rosuvastatin	0.237	0.549	5.1

Supplementary Table S1. The workflow to predict human intravitreal clearance in humans of simvastatin, atorvastatin and rosuvastatin.

Patient	Pathology	Doses (mg)	Sim	Sim acid	Atv	Atv lactone	2-OH Atv	4-OH Atv	2-OH Atv lactone	4-OH Atv lactone	Rsv	Rsv lactone	N-desmethyl Rsv
A98	PDR, TDR	10	<LOQ										
425	RRD, DR	20		<LOQ									
347	PDR, TDR	40	0.073	0.039									
370	PDR	40	0.044	0.019									
A101	PDR, TDR	40	0.027	0.006									
355	PDR	10			0.104	0.035	0.071	0.057	0.029	0.012			
352	PDR, TDR	20				0.012	0.047	0.022	0.023				
430	PDR	20			0.217	0.062	0.099	0.12	0.032	0.017			
429	RRD	20			0.053	0.016		0.022	0.023	0.008			
368	PDR	40			0.207	0.089	0.176	0.209	0.063	0.039			
383	PDR	10									0.055		
412	PDR	20									0.316	0.025	0.026
397	PDR	20									0.168	0.011	0.013
359	Pucker, diabetes	20									0.056		
428	MH, diabetes, not DR	20									0.052		
371	PDR	0*									0.241		0.019
360	PDR	0											
399	PDR, TDR	0											
405	PDR	0											
406	PDR	0											

Supplementary Table S2. The concentration (ng/ml) of simvastatin (SIM), atorvastatin (ATV), and rosuvastatin (RSV) and the corresponding metabolites from vitreous samples of patients undergoing vitrectomy, with or without (controls) oral statin treatment. Patients had four different main diagnoses: the majority were operated due to PDR (proliferative diabetic retinopathy n=16) and out of these, five patients had TRD (tractional retinal detachment), two patients were operated due to RRD (rhegmatogenous retinal detachment), one due to age-related epiretinal fibrosis i.e. pucker, and one due to macular hole (MH). LOQ: limit of quantitation.

Compound	Precursor ion m/z	Product ions m/z	Collision energy (V)	Cone Voltage (V)	Linear range (ng/ml)	Dwell time (s)	Retention time (min)
Simvastatin lactone (parent drug)	419.33	285.11	10	8	0.012-10.007	0.122	4.96
		198.84	14				
Simvastatin lactone D6	425.25	285.11	10	26		0.122	4.95
		198.84	10				
Simvastatin acid	435.25	319.13	16	40	0.005-9.854	0.122	4.51
		114.32	24				
Simvastatin acid D6	441.31	319.16	16	42		0.122	4.50
		120.36	26				
Atorvastatin (parent drug)	558.95	439.99	22	58	0.045-1.971	0.059	3.85
		249.89	40				
Atorvastatin lactone	541.27	448.25	16	8	0.011-1.974	0.185	4.12
		422.30	22				
2-OH Atorvastatin	575.28	466.28	14	62	0.047-1.946	0.080	3.14
		440.28	22				
2-OH Atorvastatin lactone	557.27	448.27	16	56	0.021-1.985	0.080	3.39
		422.26	22				
4-OH Atorvastatin	575.28	440.27	22	6	0.019-1.910	0.059	3.71
		249.91	44				
4-OH Atorvastatin lactone	557.27	448.27	18	22	0.019-1.910	0.059	3.98
		422.26	22				
Atorvastatin D5	564.33	445.31	22	54		0.059	3.84
		254.85	44				
Rosuvastatin (parent drug)	482.19	258.12	32	84	0.004-10.117	0.080	3.25
		300.19	34				
Rosuvastatin lactone	464.19	270.07	30	10	0.009-5.350	0.059	3.65
		282.07	32				
N-desmethyl Rosuvastatin lactone	468.18	243.92	34	14	0.008-10.127	0.247	2.64
		286.07	36				
Rosuvastatin D6	488.17	264.17	30	62		0.080	3.25
		306.24	38				

Supplementary Table S3. Mass spectrometry related parameters

References

1. del Amo, E. M., Vellonen, K. S., Kidron, H. & Urtti, A. Intravitreal clearance and volume of distribution of compounds in rabbits: In silico prediction and pharmacokinetic simulations

for drug development. *European Journal of Pharmaceutics and Biopharmaceutics* **95**, 215-226 (2015).

2. Reichenbach, A. *et al*. Development of the rabbit retina. V. The question of 'columnar units'. *Brain Res. Dev. Brain Res.* **79**, 72-84 (1994).

3. Panda-Jonas, S., Jonas, J. B., Jakobczyk, M. & Schneider, U. Retinal photoreceptor count, retinal surface area, and optic disc size in normal human eyes. *Ophthalmology* **101**, 519-523 (1994).
